# National trends in inpatient endometriosis admissions: Patients, procedures and outcomes, 2006−2015

**DOI:** 10.1371/journal.pone.0222889

**Published:** 2019-09-19

**Authors:** Stephanie J. Estes, Ahmed M. Soliman, Andrew J. Epstein, Julia C. Bond, Keith Gordon, Stacey A. Missmer

**Affiliations:** 1 Department of Obstetrics and Gynecology, Penn State Health, Hershey, PA, United States of America; 2 AbbVie Inc., North Chicago, IL, United States of America; 3 Medicus Economics, Philadelphia, PA, United States of America; 4 Department of Obstetrics, Gynecology and Reproductive Biology, Michigan State University, Grand Rapids, Michigan, United States of America; 5 Department of Epidemiology, Harvard T.H. Chan School of Public Health, Boston, Massachusetts, United States of America; Universita degli Studi dell'Insubria, ITALY

## Abstract

**Introduction:**

Despite guidance towards minimally invasive, outpatient procedures for endometriosis, many patients nonetheless receive inpatient care. Our objective was to assess trends in patient and hospital characteristics, surgical complications and hospital charges for women with an endometriosis-related inpatient admission in the United States.

**Methods:**

We conducted a pooled cross-sectional analysis of Healthcare Cost and Utilization Project Nationwide Inpatient Sample data. Visits were stratified into three time-period-defined cohorts (2006–2007, 2010–2011, and 2014 through the first three quarters of 2015). Visits were included if the patient was aged 18–49 years and the primary diagnosis code was for endometriosis (International Classification of Diseases, 9th Revision code 617.xx). We evaluated counts of inpatient admissions and rates of patient and hospital characteristics.

**Results:**

The number of inpatient admissions with a primary diagnosis code for endometriosis decreased by 72.8% from 2006 to 2015. At the same time, among those admitted for inpatient care for endometriosis, the proportions who had Medicaid insurance and multiple documented comorbidities increased. From 2006 to 2015, mean total hospital charges increased by 75% to $39,662 in 2015 US dollars, although average length of stay increased by <1 day.

**Conclusions:**

The number of inpatient admissions with a primary diagnosis of endometriosis decreased over the past decade, while surgical complications and associated hospital charges increased. The share of patients with multiple comorbidities increased and an increasing proportion of inpatient endometriosis admissions were covered by Medicaid and occurred at urban teaching hospitals. These findings suggest a demographic shift in patients receiving inpatient care for endometriosis towards more complex, vulnerable patients.

## Introduction

Endometriosis is a chronic inflammatory condition characterized by hormonally responsive endometrial-like tissue that exists outside of the uterus [1]. It affects 6−10% of reproductive-age women and has a considerable impact on health-related quality of life [2–5]. Endometriosis can seriously impact mental health in a variety of ways, including not only the stress of dealing with its most common symptoms (chronic pain and infertility), but also the stigma and social implications of the disease [6–8]. The pathogenesis of endometriosis remains enigmatic but certainly includes immunologic and hormonal factors, including oxidative stress, and genetic susceptibility and epigenetic modifications [6,9–11].

First-line therapies for endometriosis are usually medical management using non-steroidal anti-inflammatory drugs and estrogen-progestin combination medications [12]. Other pharmacologic treatment options include progestins, anti-progestins, gonadotropin-receptor hormone agonists or antagonists, and aromatase inhibitors [13,14]. Surgical options most often include laparoscopic excision or ablation of lesions, hysterectomy, and/or oophorectomy [14]. Choice of surgical approach is influenced by patient preferences, family planning status, and disease severity [14]. Often a combination of medical and surgical management is necessary [14,15].

Minimally invasive surgical options have been broadly recommended to reduce the burden on patients and the healthcare system [14–17]. Slow but steady response to this recommendation is evident; for example, the share of hysterectomies performed laparoscopically increased from 10% in 1997 to 29% in 2010 and 43% in 2013 [18–20]. A recent study of the American College of Surgeons National Surgical Quality Improvement Program database found that 30% of recorded hysterectomies for benign indications in 2015 were abdominal, while 16% were vaginal and 55% were laparoscopic [21]. The move towards minimally invasive gynecologic surgery has led to a greater proportion of outpatient procedures, shorter hospital stays, fewer complications, and faster recovery for patients [22], though inpatient surgery remains necessary for some patients.

Despite evidence that the number of inpatient gynecological procedures performed in the United States has decreased in recent years, little is known about the patients still receiving inpatient care for endometriosis. Accordingly, we evaluated patterns of inpatient care for endometriosis using a recent, nationally representative sample of inpatient admissions in the United States. We analyzed trends in resource use, outcomes, and patient characteristics to obtain a more complete picture of inpatient care even as treatment recommendations have shifted towards outpatient management.

## Materials and methods

### Data source

This pooled cross-sectional study used data from the Health Care Utilization Project National Inpatient Sample (NIS) collected by the Agency for Healthcare Research and Quality. The NIS is a publicly available data source that contains a random sample of discharges from all non-federal hospitals in the United States. It captures approximately 20% of all community-hospital discharges in the US and when weighted represents an estimated 35 million hospitalizations annually [23]. It relies on administrative billing records to collect information on patient demographic and insurance characteristics, geographic location, International Classification of Diseases, Ninth Revision, Clinical Modification (ICD-9-CM) diagnosis and procedure codes, total charges, and hospital characteristics. NIS data were collected based on a complex sampling design. Prior to 2012, annual stratified random samples of hospitals were identified, and 100% of discharges were collected from these hospitals. In 2012, the NIS design changed to sampling at the discharge level [24]. NIS data are publicly available and completely de-identified. Thus, their use does not constitute Human Subjects Research under the United States Federal Policy for the Protection of Human Subjects (the Common Rule) 45 CFR part 46, and consideration of this study by an Institutional Review Board is not required.

### Sample

We obtained data from three mutually exclusive time periods that defined our cohorts: 2006–2007, 2010–2011, and 2014 through the third quarter of 2015, after which the NIS switched from ICD-9-CM to International Classification of Diseases, Tenth Revision, Clinical Modification (ICD-10-CM) codes. Inpatient admissions were identified with a principal diagnosis code of endometriosis (ICD-9-CM codes 617.xx). Women were excluded if they were younger than 18 years or older than 49 years old at admission, or if they had a diagnosis code for malignant neoplasms of female genital organs (ICD-9-CM codes 179 and 180.0–184.9), to increase the likelihood that observed procedures were being performed strictly for endometriosis management.

### Variables

Variables of interest included patient and hospital characteristics for each time-period-defined cohort. We also assessed the prevalence of 29 Elixhauser chronic comorbid conditions, which are commonly used to measure disease burden and case mix in hospital discharge data [25]. We calculated the prevalence of seven surgical procedures commonly associated with endometriosis management across time-period-defined cohorts: hysterectomy, laparoscopy, laparotomy, oophorectomy, bladder interventions, salpingectomy, and other excisions/ablations (see S1 Table for a list of procedures and their codes). Finally, we evaluated trends in surgical complications (see S2 Table for a list of complications and their codes), length of inpatient hospital stay, and total hospital charges. Total charges were inflated to 2015 US dollars using the Personal Health Care Price Index for Hospital Care [26].

### Analyses

Descriptive statistics for each time-period-defined cohort were calculated as counts and percentages for categorical variables and means for continuous variables. Unadjusted proportions and means were compared across time-period-defined cohorts using Pearson chi-squared tests or ANOVA tests. Missing values were reported as a separate category where relevant. All analyses were weighted to provide nationally representative estimates. For data prior to 2012, we used the NIS sample trend weights [27], whereas for subsequent years we used the cross-sectional weights. The weighting produced a large sample size in addition to nationally representative estimates; thus, results should be interpreted in terms of clinical as well as statistical significance.

## Results

**Fig 1:**
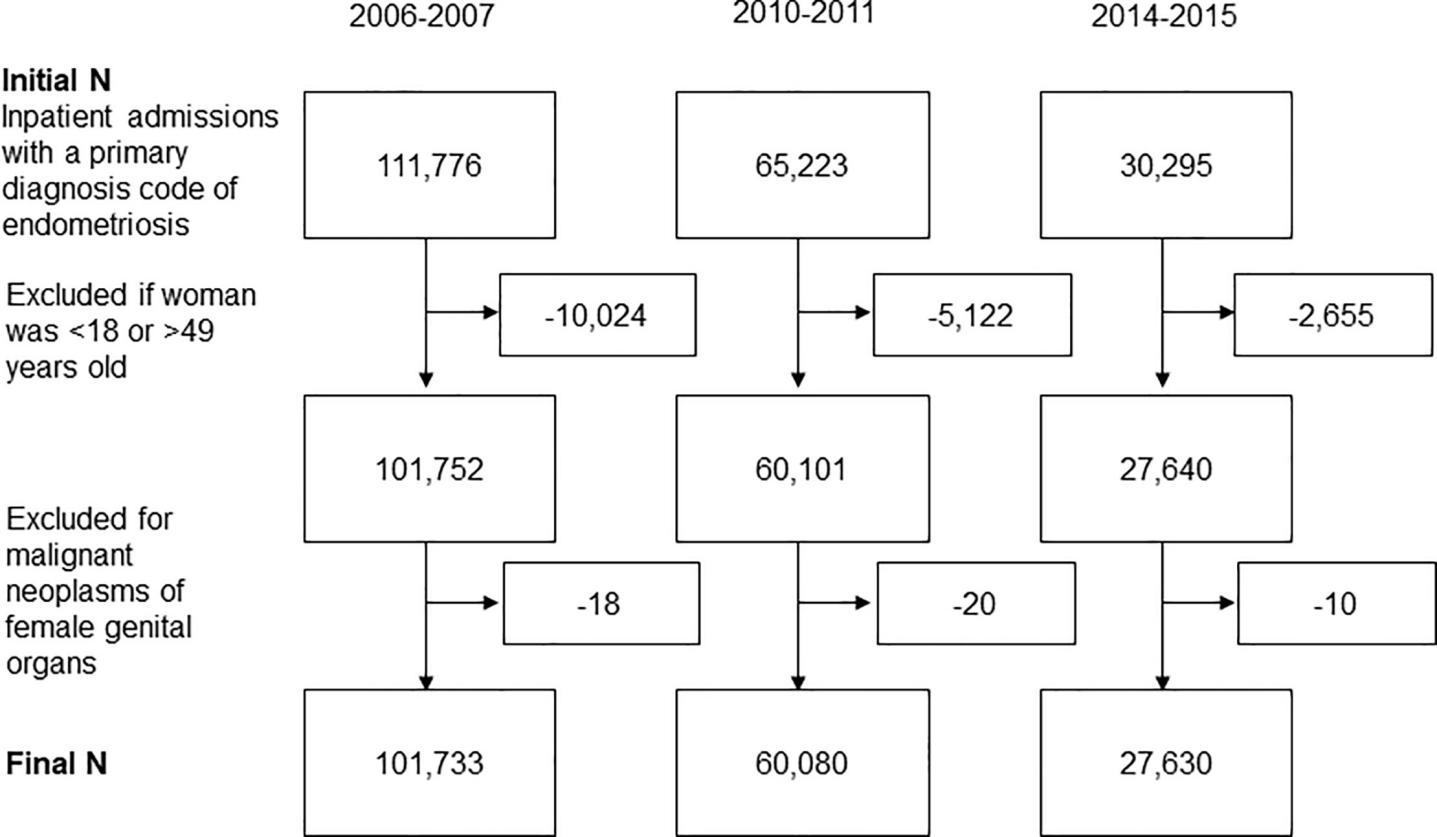
Sample selection protocol. Sample sizes are based on weighted national estimates; numbers may not add due to rounding.

For women with endometriosis as identified by an ICD-9-CM diagnosis code, the final sample consisted of 101,733 inpatient admissions for the 2006–07 cohort, 60,080 for the 2010–11 cohort, and 27,630 for the 2014–15 cohort (Fig 1).

Patient and hospital characteristics are presented by time-period-defined cohort in Table 1.

**Table 1:** Patient and hospital characteristics of women with an inpatient admission for endometriosis (ICD-9 code 617.xx) within time-period-defined cohorts.

	Cohort (defined by years)			
	2006–07	2010–11	2014–15*	P-Value^†^
Weighted Sample Size^‡^	101,733	60,080	27,630	
	(%) of total cohort	(%) of total cohort	(%) of total cohort	
Variable				
Age				
18–29 Years	13.4%	14.2%	14.2%	0.03
30–39 Years	40.4%	41.6%	41.5%	
40–49 Years	46.1%	44.2%	44.3%	
Payer Type			
Private	79.3%	71.8%	66.1%	<0.001
Medicaid	10.7%	15.6%	22.0%	
Uninsured	3.3%	4.7%	4.3%	
Medicare and Other	6.5%	7.7%	7.3%	
Missing	0.2%	0.2%	0.2%	
Elixhauser Comorbidity^§^ Count				
Zero	59.9%	53.8%	48.8%	<0.001
One	26.0%	27.2%	28.4%	
Two	9.9%	12.2%	13.9%	
Three or More	4.2%	6.8%	8.9%	
National Quartile of ZIP Code-level Median Household Income^‖^				
First	21.4%	22.7%	26.6%	0.005
Second	26.7%	24.8%	25.9%	
Third	26.4%	27.8%	24.6%	
Fourth	23.6%	23.0%	21.0%	
Missing	1.9%	1.7%	1.8%	
Race/Ethnicity^¶^				
White Non-Hispanic	50.5%	60.5%	57.7%	<0.001
Black Non-Hispanic	6.2%	10.1%	13.8%	
Hispanic	7.3%	10.6%	14.7%	
Other Non-Hispanic	4.5%	6.9%	8.7%	
Missing/Not Reported	31.5%	11.9%	5.1%	
Hospital Census Region				
Northeast	13.5%	14.8%	17.3%	0.06
Midwest	25.8%	23.1%	21.6%	
South	38.7%	37.8%	37.4%	
West	22.0%	24.3%	23.8%	
Hospital Bed Size Category^#^				
Small	13.8%	11.3%	16.9%	<0.001
Medium	25.0%	25.8%	30.7%	
Large	61.1%	61.7%	52.4%	
Missing	0.1%	1.2%	0.0%	
Hospital Location/Teaching Category				
Rural	13.1%	13.1%	11.1%	<0.001
Urban-NonTeaching	46.2%	42.7%	25.2%	
Urban-Teaching	40.6%	43.0%	63.7%	
Missing	0.1%	1.2%	0.0%	

Abbreviation: ICD-9: International Classification of Diseases, 9^th^ edition

* Data for 2015 cover Q1-Q3 only.

^†^ P-values are from Pearson chi-squared tests that account for survey design.

^‡^ Weighted counts are nationally representative.

^**§**^ Elixhauser comorbidities are based on a roster of 29 comorbidities using ICD-9 diagnosis codes.^23^

^**‖**^ The first quartile corresponds to ZIP Codes with median household income below the 25^th^ percentile; the fourth corresponds to ZIP Codes above the 75^th^ percentile.

^¶^ The HCUP NIS combines race and Hispanic ethnicity into a single measure.

^**#**^ Hospital bed-size categories were defined differently based on hospital geographic location, urban/rural, and teaching/non-teaching designation (see: https://www.hcup-us.ahrq.gov/db/vars/hosp_bedsize/nisnote.jsp).

**Fig 2:**
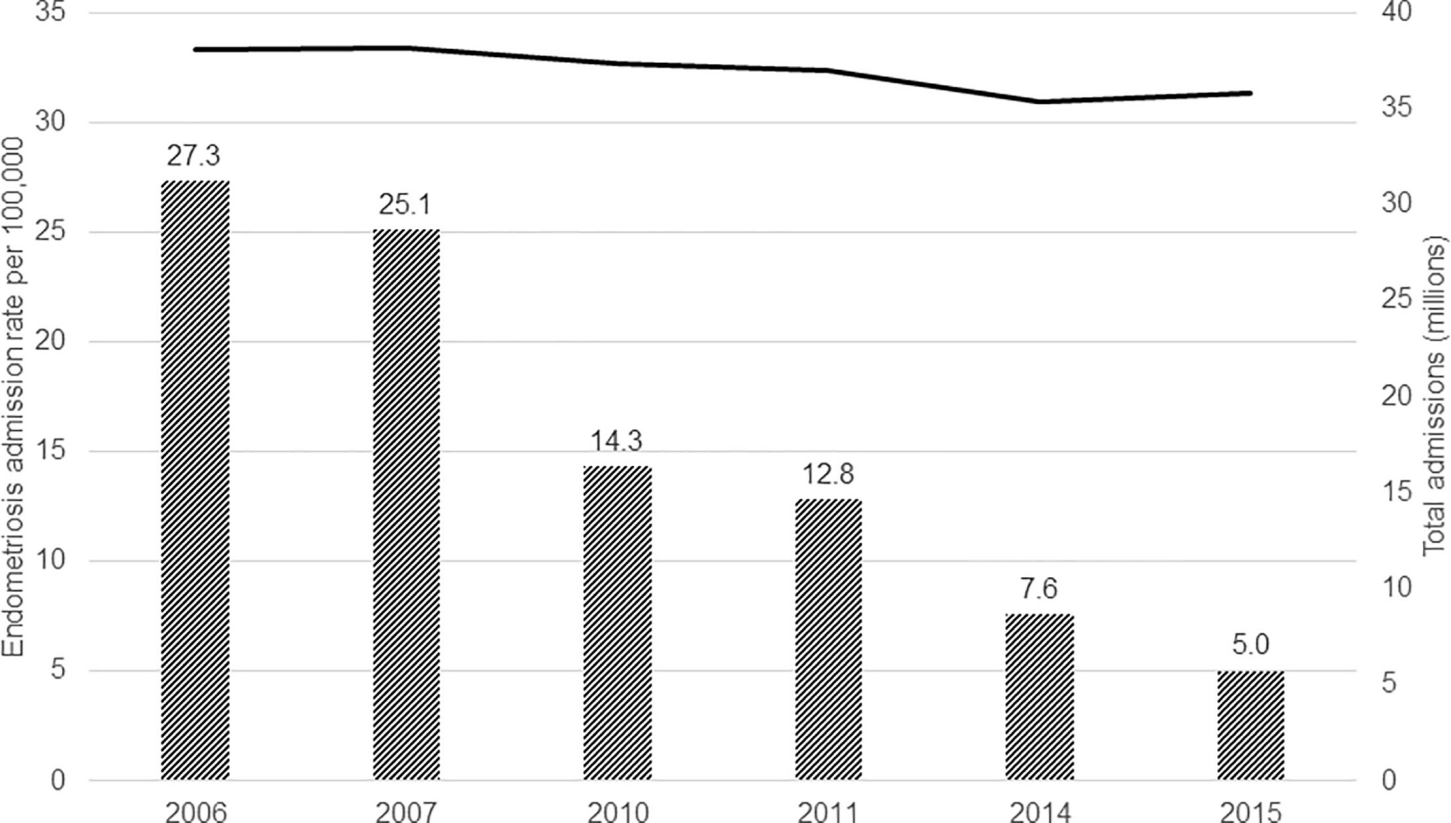
Nationally representative endometriosis inpatient admission rates and total inpatient admissions trends, 2006–2015. Bars represent the annual endometriosis-related inpatient admission rate per 100,000; solid line represents total inpatient admissions in millions.

Overall, the number of inpatient admissions for endometriosis decreased over the 10-year period studied and did so at a faster rate than inpatient admissions for all conditions, which were essentially flat at between 35 and 38 million admissions (Fig 2). There were also notable changes in the composition of patient and hospital characteristics of inpatient admissions for endometriosis. A higher percentage of inpatient admissions for endometriosis had Medicaid insurance coverage in the 2014–15 cohort compared to 2006–07 (22.0% vs 10.7%). Further, the proportion of women receiving endometriosis-related inpatient care who lived in ZIP Codes with a median income in the lowest quartile nationally increased over time, although more modestly (from 21.4% in 2006–07 to 26.6% in 2014–15). Patient comorbidity burden also increased over time. In 2006–07, 59.9% of admissions had no Elixhauser comorbidities compared with 48.4% in 2014–15. The percentage of admissions with ≥ 3 comorbidities doubled from 2006–07 to 2014–15 (4.2% vs 8.9%).

There were also changes in the characteristics of hospitals at which inpatient admissions for endometriosis occurred. In 2014–15, most patients were admitted to urban-teaching hospitals (62.7% in 2014–15 vs. 40.6% in 2006–07 and 43.0% in 2010–11; p<0.001). Table 2 describes the prevalence of Elixhauser comorbidities within the three time-period-defined cohorts. There were differences in the prevalence of many comorbidities across cohorts, including hypertension (10.6%, 12.5%, and 14.7% for 2006–07, 2010–11, and 2014–15, respectively, p<0.001) and drug abuse (0.5%, 1.0% and 1.3% for 2006–07, 2010–11, and 2014–15, respectively, p<0.001).

**Table 2:** Trends in the prevalence of comorbidities among women with an inpatient admission for endometriosis (ICD-9 code 617.xx) within time-period-defined cohorts.

	Cohort (defined by years)				
	2006–07	2010–11	2014–15*	P-Value for 2006–07 vs 2010–11 vs 2014–15^†^	P-Value for 2006–07 vs 2014–15^†^
Weighted Sample Size^‡^	101,733	60,080	27,630		
AHRQ Elixhauser Comorbidities^**§**^	(%) of total cohort	(%) of total cohort	(%) of total cohort		
Congestive heart failure	0.1%	0.1%	0.3%	0.009	0.002
Valvular disease	1.9%	1.3%	0.9%	<0.001	<0.001
Pulmonary circulation disorders	0.1%	0.2%	0.2%	0.41	0.30
Peripheral vascular disorders	0.1%	0.1%	0.2%	0.14	0.08
Hypertension	10.6%	12.5%	14.7%	<0.001	<0.001
Paralysis	0.1%	0.2%	0.2%	0.61	0.70
Other neurological disorders	1.4%	1.8%	2.4%	<0.001	<0.001
Chronic pulmonary disease	8.1%	9.6%	10.3%	<0.001	<0.001
Diabetes, uncomplicated	3.2%	4.0%	4.7%	<0.001	<0.001
Diabetes with chronic complications	0.1%	0.2%	0.3%	0.03	0.010
Hypothyroidism	4.9%	5.9%	6.4%	<0.001	<0.001
Renal failure	0.2%	0.2%	0.5%	<0.001	<0.001
Liver disease	0.3%	0.4%	0.6%	<0.001	<0.001
Peptic ulcer disease excluding bleeding	0.004%	0.02%	0%	0.35	0.60
Acquired immune deficiency syndrome	0.02%	0.04%	0.02%	0.43	0.97
Lymphoma	0.05%	0.01%	0.04%	0.19	0.77
Metastatic cancer	0.04%	0.03%	0.1%	0.79	0.55
Solid tumor without metastasis	0.2%	0.2%	0.2%	0.57	0.33
Rheumatoid arthritis/collagen vascular diseases	0.9%	1.2%	1.3%	0.008	0.010
Coagulopathy	0.4%	0.5%	0.9%	<0.001	<0.001
Obesity	6.10%	9.4%	13.3%	<0.001	<0.001
Weight loss	0.1%	0.3%	0.5%	<0.001	<0.001
Fluid and electrolyte disorders	1.8%	3.0%	3.6%	<0.001	<0.001
Chronic blood loss anemia	3.3%	2.1%	2.2%	<0.001	<0.001
Deficiency anemias	6.2%	8.2%	10.4%	<0.001	<0.001
Alcohol abuse	0.1%	0.3%	0.3%	0.005	0.005
Drug abuse	0.5%	1.0%	1.3%	<0.001	<0.001
Psychoses	1.5%	2.2%	2.6%	<0.001	<0.001
Depression	7.3%	10.2%	9.9%	<0.001	<0.001

Abbreviations: ICD-9: International Classification of Diseases, 9^th^ edition; AHRQ: Agency for Healthcare Research and Quality

* Data for 2015 cover Q1-Q3 only.

^†^ P-values are from ANOVA tests of equality that accounted for survey design.

^‡^ Weighted counts are nationally representative.

^**§**^ Elixhauser comorbidities were constructed using ICD-9 diagnosis codes.

There were also changes in the frequency of endometriosis-associated surgical procedures across the decade (Table 3). The proportion of inpatient admissions that involved a hysterectomy changed from 78.2% in 2006–07 to 67.4% in 2014–15 (p<0.001). The prevalence of laparoscopy remained at approximately 3% in all three time periods. Overall, the proportion of inpatient admissions with a primary diagnosis of endometriosis that included any of the seven endometriosis-related surgeries decreased over time, from 95.5% in 2006–07 to 91.1% in 2014–15. Among those without an endometriosis-related surgery of interest, over the full study period 46% had no surgery and 54% had another type of surgery.

**Table 3:** Trends in surgical procedures reported in inpatients visits for endometriosis across cohorts.

	Cohort (defined by years)				
Outcome	2006–07	2010–11	2014–15*	P-Value for 2006–07 vs 2010–11 vs 2014–15^†^	P-Value for 2006–07 vs 2014–15^†^
Weighted Sample Size^‡^	101,733	60,080	27,630		
Surgical Procedure^§^	(%) of total cohort	(%) of total cohort	(%) of total cohort		
Hysterectomy	78.2%	74.1%	67.4%	<0.001	<0.001
Bladder Intervention	0.4%	0.5%	0.4%	0.98	0.93
Laparoscopy	2.5%	2.4%	3.0%	0.06	0.04
Laparotomy	11.2%	11.0%	13.1%	0.004	0.002
Oophorectomy	62.9%	60.1%	58.3%	<0.001	<0.001
Other Excision/Ablation	8.2%	10.2%	11.9%	<0.001	<0.001
Salpingectomy	1.0%	1.5%	2.7%	<0.001	<0.001
Any of the Above Surgeries	95.9%	93.9%	91.1%	<0.001	<0.001

* Data for 2015 cover Q1-Q3 only.

^†^ P-values are from ANOVA tests of equality that accounted for survey design.

^‡^ Weighted counts are nationally representative.

^**§**^ These seven categories of surgical procedures were selected as the most common surgical procedure types used for endometriosis management.

Table 4 reports trends in surgical complications by surgery type, length of inpatient stay, and total hospital charges. The prevalence of complications increased over time following hysterectomy, laparoscopy, and oophorectomy. While length of inpatient stay increased by <1 day, there was a large increase in total charges (in 2015 USD) from $22,642 in 2006–2007 to $39,662 in 2014–2015 (p<0.001).

**Table 4:** Trends in surgical complications, total charges and inpatient length of stay across cohorts.

	Cohort (defined by years)				
Outcome	2006–07	2010–11	2014–15*	P-Value for 2006–07 vs 2010–11 vs 2014–15^†^	P-Value for 2006–07 vs 2014–15^†^
Weighted Sample Size^‡^	101,733	60,080	27,630		
	(%) of total cohort	(%) of total cohort	(%) of total cohort		
Prevalence of Complications by Surgical Procedure^§^						
Hysterectomy	12.4%	13.3%	15.3%	<0.001	<0.001
Bladder Intervention	12.1%	10.2%	17.4%	0.61	0.47
Laparoscopy	15.2%	17.6%	24.0%	0.053	0.016
Laparotomy	15.3%	16.8%	19.9%	0.02	0.005
Oophorectomy	13.7%	14.3%	16.6%	0.001	<0.001
Other Excision/Ablation	17.0%	16.8%	18.7%	0.59	0.38
Salpingectomy	17.6%	16.1%	12.8%	0.46	0.21
Any of the Above Surgeries	12.9%	13.8%	16.0%	<0.001	<0.001
Mean Length of Stay (Days)	2.4	2.3	2.6	<0.001	0.013
Mean Total Charges per Stay (2015 USD)	22,642	30,977	39,662	<0.001	<0.001

Abbreviation: USD: United States Dollars

* Data for 2015 covers Q1-Q3 only.

^†^ P-values are from ANOVA tests of equality that accounted for survey design.

^‡^ Weighted counts are nationally representative.

^**§**^ These seven categories of surgical procedures were selected as the most common surgical procedure types used for endometriosis management.

## Discussion

### Principal findings

During 2006–2015 the number of inpatient admissions for endometriosis decreased. Although hysterectomy and oophorectomy remained the predominant inpatient surgical procedures observed, their prevalence declined slightly. The prevalence of surgical complications increased overall and by procedure type. Average length of stay increased slightly, and total hospital charges increased considerably.

The composition of women being admitted for endometriosis changed over the same period. Higher proportions of women in later cohorts had multiple comorbidities, were covered by Medicaid as opposed to commercial insurance, and lived in lower-income ZIP Codes. This raises the possibility that, as inpatient admissions for endometriosis have become less common, women who are admitted for endometriosis have fewer resources and are at higher risk than women who receive care in other, presumably outpatient, settings.

Prior research suggests women with fewer resources and more comorbidities are more likely to receive a hysterectomy [28]. Further, there is compelling evidence that patients with higher income, private insurance, and white race are more likely to undergo laparoscopic hysterectomy versus open hysterectomy [19,21,29]. These trends are increasingly visible in inpatient endometriosis care. This is especially concerning as endometriosis-associated symptoms can recur following hysterectomy [30]; thus, vulnerable patients may incur higher risks for procedures that do not confer higher benefits.

Increased complications and patient comorbidities may have helped drive the increases in hospital charges. Between 2003 and 2014 the percentage of inpatient admissions with multiple chronic conditions increased from 64% to 78%, with the fastest growth among patients aged 18–44 years [31]. This suggests that patient complexity continues to be an important consideration when evaluating inpatient care, especially regarding associated resource use and costs.

### Clinical implications

Our study draws attention to an important population of patients: those who continued to receive inpatient care for endometriosis even as management guidelines emphasized minimally invasive care. It is important to monitor this population of women to ensure that women who receive inpatient treatment are only those who are indicated for it, to guard against disparities based on non-clinical factors such as income, education, geographic location, or race/ethnicity. It is also important to assess the population currently requiring inpatient care to inform efforts to reduce the need for inpatient care in the future, indeed even among women for whom it may be indicated today.

Additionally, there are important economic implications to our study that are relevant to clinical care. Given that the associated mean total charges per stay increased, inpatient care—particularly surgical care—remains an important contributor to the overall economic burden of endometriosis. Total costs of surgery for endometriosis are high and include complications, medical management during recovery and follow-up, and the possibility of retreatment in addition to the cost of the procedure [32,33]. Endometriosis-related surgery is also associated with considerable indirect costs in the form of increased absenteeism and presents a significant economic burden to society [34]. Inpatient costs, and the economic burden of endometriosis overall, may continue to rise if vulnerable patients, including those with multiple comorbidities, are preferentially directed towards invasive procedures requiring inpatient care.

### Research implications

Our findings add an important and objective assessment of national trends in the still sizeable number of inpatient procedures that occur for endometriosis even as guidelines recommend outpatient care settings when possible. Current outpatient medical management of endometriosis may also affect inpatient care and deserves further study. As the number of inpatient admissions related to endometriosis decreased over time, patients who received inpatient care for endometriosis were increasingly at higher risk and more disadvantaged. This may be indicative of the lack of access of vulnerable populations to timely and effective medical management or outpatient surgical care, though this would need to be confirmed with a corresponding analysis of outpatient data. Our study also provides an important characterization of the comorbidities associated with patients who are receiving inpatient care for endometriosis, which could be important information for hospitals seeking to improve inpatient endometriosis care. Finally, we observed contemporaneous increases in hospital charges and length of stay as well as surgical complications. The growth in hospital charges over 2006–2015 continues an earlier rising trend reported in an analysis of HCUP NIS data for 1993–2002; in contrast, while declining from 1993 to 2000, length of stay has not changed much since then [35]. Limitations in the currently available data preclude us from going beyond describing the national trends; future research is needed to explore potential explanations.

### Strengths and limitations

Our study used the NIS database, which consists of a random sample of non-federal hospitals that, when weighted, produces a nationally representative estimate of US inpatient admissions. The NIS database has several limitations, however. It includes only individuals who received inpatient healthcare services. Also, it does not follow individual women over time or cover healthcare use outside of inpatient hospitalizations. Thus, the full scope of endometriosis-related care, including outpatient procedures, is not captured. The study period ended in 2015, and practice patterns may have changed since then, although many of the changes observed were modest across the decade captured in this study. Only the first three quarters of 2015 were analyzed because of the change to ICD-10-CM coding and the associated risk of diagnosis and procedure misclassification [36].

Moreover, the NIS database, as is true of all large ICD-based data capture, is limited because it is based on administrative data that are collected primarily for billing and reimbursement. It offers limited detail about clinical decision-making, patient complexity, and other important aspects of the clinical encounter. The NIS data may suffer from coding inaccuracies as well as inconsistencies across hospitals. There are likely missed endometriosis-related procedures, particularly when endometriosis was present but not coded. It is also possible that patients are being incorrectly diagnosed with endometriosis, particularly if diagnoses are based on clinical symptoms. These coding patterns can vary informatively by clinician specialty and patient and hospital characteristics. In addition, the NIS reports hospital charges, which represent neither reimbursed amounts nor the costs faced by hospitals.

Of particular importance to the study of endometriosis, the NIS does not contain information about endometriosis stage nor certain details about the surgical procedures performed in terms of the intensity of dissection required. Unfortunately, given a lack of standardization of surgical documentation, it is currently not possible to abstract this information from any form of medical records across multiple practitioners within or among surgical sites [37]. All forms of endometriosis are challenging to treat; however, surgical complexity and requirement for advanced expertise is particularly high for deep endometriosis. Techniques such as superficial and deep rectal shaving and nerve sparing intestinal dissection are important considerations [38–41]. Future studies will require standardized collection of these endometriosis phenotypic presentation and surgical approach details to parse out adequately whether there has been shift over time in inpatient procedures toward women with more surgically complex endometriosis presentation that may have previously been mis- or undiagnosed.

An obvious restriction in scope of our analysis is that it examines endometriosis surgical treatment only in the inpatient setting; the inpatient setting is the focus of our review to allow characterization of this population. These findings should not be generalized to those receiving care in the outpatient surgery setting. Endometriosis treatment recommendations have focused on increasing the use of outpatient surgery (for example, laparoscopic lesion ablation/excision, ovarian cystectomy for endometriomas, and lysis of adhesions [42]), and recent data suggest that outpatient surgery has become commonplace. For 29 states with administrative data on all inpatient admissions and ambulatory surgery visits, 78.0% of endometriosis cases in 2014 were treated in an ambulatory surgery setting [43]. Unfortunately, there is no nationally representative database of outpatient visits corresponding to the HCUP NIS that we could use to assess treatment patterns overall and compare trends in treatment patterns between care settings. There are several possible explanations for our observed trends in inpatient management, including case selection for inpatient surgery to those with more complex presentation, associated comorbidities that do not allow the patient to receive care at an outpatient surgery center (e.g., body mass index), or changes in documentation such as altered ICD code attribution. Because there is no corresponding outpatient national dataset available, we are unable to explore which factors may be contributing to our observations.

### Conclusions

Surgery is an important clinical option for endometriosis, and it remains central to classifying the disease [37]. However, minimally invasive approaches should be prioritized. Because of its serious personal and societal implications, tracking clinical outcomes, economic burden and health disparities associated with endometriosis is critical to ensure that all women have access to appropriate treatment.

## Supporting information

S1 Table Surgical procedures and corresponding ICD-9 diagnosis and procedure codes included in the analysis. (DOCX)

S2 Table Surgical complications and corresponding ICD-9 diagnosis and procedure codes included in the analysis. (DOCX)

## References

[1] Giudice LC. Endometriosis. N Engl J Med. 2010 6 24;362(25):2389–98. 10.1056/NEJMcp1000274 20573927 PMC3108065

[2] Fuldeore MJ, Soliman AM. Prevalence and symptomatic burden of diagnosed endometriosis in the United States: national estimates from a cross-sectional survey of 59,411 women. Gynecol Obstet Inves 2017;82(5):453–61. 10.1159/000452660 27820938

[3] Eskenazi B, Warner ML. Epidemiology of endometriosis. Obstet Gyn Clin N Am 1997;24(2):235–58. 10.1016/s0889-8545(05)70302-8 9163765

[4] Missmer SA, Hankinson SE, Spiegelman D, Barbieri RL, Marshall LM, Hunter DJ. Incidence of laparoscopically confirmed endometriosis by demographic, anthropometric, and lifestyle factors. Am J Epidemiol. 2004 10 15;160(8):784–96. 10.1093/aje/kwh275 15466501

[5] Louis GM, Hediger ML, Peterson CM, Croughan M, Sundaram R, Stanford J, et al Incidence of endometriosis by study population and diagnostic method: the ENDO study. Fertil Steril. 2011 8 1;96(2):360–5. 10.1016/j.fertnstert.2011.05.087 21719000 PMC3143230

[6] Laganà AS, La Rosa VL, Rapisarda AMC, Valenti G, Sapia F. Chiofalo B. Anxiety and depression in patients with endometriosis: impact and management challenges. Int J Womens Health. 2017;9:323–330. 10.2147/IJWH.S119729 28553145 PMC5440042

[7] Facchin F, Barbara G, Dridi D, Alberico D, Buggio L, Somigliana E, Saita E, Vercellini P. Mental health in women with endometriosis: searching for predictors of psychological distress. Hum Reprod. 2017;32(9):1855–1861. 10.1093/humrep/dex249 28854724

[8] Smorgick N, As-Sanie S. Pelvic pain in adolescents. Semin Reprod Med. 2018;36(2):116–122. 10.1055/s-0038-1676088 30566977

[9] Vitale SG, Capriglione S, Peterlunger I, La Rosa VL, Vitagliano A, Noventa M, et al The role of oxidative stress and membrane transport systems during endometriosis: a fresh look at a busy corner. Oxid Med Cell Longev. 2018 10.1155/2018/7924021. PMC5883985 29743986

[10] Dordts S, Koninckx P, Brosens I. Pathogenesis of deep endometriosis. Fertil Steril. 2017;108(6):872–885. 10.1016/j.fertnstert.2017.08.036 29100623

[11] Zondervan KT, Becker CM, Koga K, Missmer SA, Taylor RN, Vigano P. Endometriosis. Nat Rev Dis Primers. 2018;4(1):9 10.1038/s41572-018-0008-5 30026507

[12] Falcone T, Flyckt R. Clinical management of endometriosis. Obstet Gynecol 2018;131(3):557–71. 10.1097/AOG.0000000000002469 29420391

[13] Gambone JC, Mittman BS, Munro MG, Scialli AR, Winkel CA, Chronic Pelvic Pain/Endometriosis Working Group. Consensus statement for the management of chronic pelvic pain and endometriosis: proceedings of an expert-panel consensus process. Fertil Steril. 2002 11 1;78(5):961–72. 10.1016/s0015-0282(02)04216-4 12413979

[14] Dunselman GAJ, Vermeulen N, Becker C, Calhaz-Jorge C, D'Hooghe T, De Bie B, et al ESHRE guideline: management of women with endometriosis. Hum Reprod. 2014 3 1; 29(3):400–12. 10.1093/humrep/det457 24435778

[15] Practice Committee of the American Society for Reproductive Medicine. Endometriosis and infertility: a committee opinion. Fertil Steril. 2012 9 1;98(3):591–98. 10.1016/j.fertnstert.2012.05.031 22704630

[16] Vercellini P, Frattaruolo MP, Buggio L. Toward minimally disruptive management of symptomatic endometriosis: reducing low-value care and the burden of treatment. Expert Rev Pharm Out. 2018 12 6;18(1):1–4. 10.1080/14737167.2018.1411803 29186995

[17] National Institute for Health Care Excellence. Endometriosis: diagnosis and management [Internet]; 2017 [cited 2019 May 9]. Available from: https://www.nice.org.uk/guidance/ng73/chapter/Recommendations#surgical-management.

[18] Farquhar CM, Steiner CA. Hysterectomy rates in the United States 1990–1997. Obstet Gynecol. 2002 2 1;99(2):229–34. 10.1016/s0029-7844(01)01723-9 11814502

[19] Lee J, Jennings K, Borahay MA, Rodriguez AM, Kilic GS, Snyder RR, et al Trends in the national distribution of laparoscopic hysterectomies from 2003 to 2010. J Minim Invas Gyn. 2014 7 1;21(4):656–61. 10.1016/j.jmig.2014.01.012 PMC4318237 24462854

[20] Morgan DM. Kamdar NS, Swenson CW, Kobernik EK, Sammarco AG, Nallamothu B. Nationwide trends in the utilization of and payments for hysterectomy in the United States among commercially insured women. Am J Obstet Gynecol. 2018 4 1; 218(4):425–e1. 10.1016/j.ajog.2017.12.218 29288067 PMC5931386

[21] Alexander AL, Strohl AE, Rieder S, Holl J, Barber EL. 2019. Examining disparities in route of surgery and postoperative complications in black race and hysterectomy. Obstet Gynecol. 2019 1 1;133(1):6–12. 10.1097/AOG.0000000000002990 30531569 PMC6326082

[22] Walsh CA, Walsh SR, Tang TY, Slack M. Total abdominal hysterectomy versus total laparoscopic hysterectomy for benign disease: a meta-analysis. Eur J Obstet Gyn R B. 2009 May 1;144(1):3–7. 10.1016/j.ejogrb.2009.01.003 19324491

[23] HCUP Databases. Healthcare Cost and Utilization Project (HCUP) [Internet]. 8 2018 Rockville, MD: US Agency for Health Care Research and Quality [cited May 9, 2019]. Available from: www.hcup-us.ahrq.gov/nisoverview.jsp.

[24] Houchens R, Ross D, Elixhauser A, Jiang J. Nationwide Inpatient Sample (NIS) Redesign Final Report. 2014 HCUP Methods Series Report # 2014–04 ONLINE. 4 4, 2014 Rockville, MD: US Agency for Health Care Research and Quality [cited May 9, 2019]. Available from: https://www.hcup-us.ahrq.gov/reports/methods/2014-04.pdf.

[25] Elixhauser A, Steiner C, Harris DR, Coffey RM. Comorbidity measures for use with administrative data. Med Care. 1998;36(1)8–27. 10.1097/00005650-199801000-00004 9431328

[26] Using appropriate price indices for analyses of health care expenditures or income across multiple years Rockville, MD: US Agency for Health Care Research and Quality [cited May 9, 2019]. Available from: https://meps.ahrq.gov/about_meps/Price_Index.shtml.

[27] Houchens RL, Ross D, Elixhauser A. Using the HCUP National Inpatient Sample to Estimate Trends. 2015. HCUP Methods Series Report # 2006–05 ONLINE. 1 4, 2016 Rockville, MD: US Agency for Health Care Research and Quality [cited May 9, 2019]. Available from: https://www.hcup-us.ahrq.gov/reports/methods/2006_05_NISTrendsReport_1988-2004.pdf.

[28] Howard BV, Kuller L, Langer R, Manson JE, Allen C, Assaf A, et al Risk of cardiovascular disease by hysterectomy status, with and without oophorectomy: The Women’s Health Initiative Observational Study. Circulation. 2005 3 29;111(12)1462–70. 10.1161/01.CIR.0000159344.21672.FD 15781742

[29] Cohen SL, Vitonis AF, Einarsson JI. Updated hysterectomy surveillance and factors associated with minimally invasive hysterectomy. JSLS-J Soc Laparoend 2014 7;18(3): e2014.00096. 10.4293/JSLS.2014.00096 PMC4208898 25392662

[30] Rizk B, Fischer AS, Lotfy HA, Turki R, Zahed HA, Malik R, et al Recurrence of endometriosis after hysterectomy. Facts Views Vis Obgyn. 2014;6(4)219–27. 25593697 PMC4286861

[31] Steiner CA, Barrett ML, Weiss AJ, Andrews RM. Trends and projections in hospital stays for adults with multiple chronic conditions, 2003–2014 HCUP Statistical Brief #183. Rockville, MD: Agency for Health Care Research and Quality [cited May 9, 2019]. Available from: http://www.hcup-us.ahrq.gov/reports/statbriefs/sb183-Hospitalizations-Multiple-Chronic-Conditions-Projections-2014.pdf. 25558764

[32] Surrey ES Soliman AM, Yang H, Du EX, Su B. Treatment patterns, complications and health care utilization among endometriosis patients undergoing a laparoscopy or a hysterectomy: a retrospective claims analysis. Adv Ther. 2017 Nov 1;34(11):2436–2451. 10.1007/s12325-017-0619-3 29039055 PMC5702365

[33] Soliman AM, Du EX, Yang H, Wu EQ, Haley JC. Retreatment rates among endometriosis patients undergoing hysterectomy or laparoscopy. J Womens Health. 2017 6 1;26(6):644–54. 10.1089/jwh.2016.6043 28472602

[34] Soliman AM, Taylor H, Bonafede M, Nelson JK, Castelli-Haley J. Incremental direct and indirect cost burden attributed to endometriosis surgeries in the United States. Fertil Steril. 2017 5 1;107(5):1181–1190. 10.1016/j.fertnstert.2017.03.020 28476181

[35] Gao X, Outley J, Botteman M, Spalding J, Simon JA, Pashos CL. Economic burden of endometriosis. Fertil Steril. 2006 12 1;86(6):1561–72. 10.1016/j.fertnstert.2006.06.015 17056043

[36] Elixhauser A, Heslin KC, Owens PL. Healthcare Cost and Utilization Project (HCUP) Recommendations for Reporting Trends Using ICD-9-CM and ICD-10-CM/PCS Data. ONLINE. Revised July 5, 2017. Rockville, MD: US Agency for Health Care Research and Quality [cited May 9, 2019]. Available from: https://www.hcup-us.ahrq.gov/datainnovations/HCUP_RecomForReportingTrends_070517.pdf. Retrieved April 5, 2019.

[37] Johnson NP, Hummelshoj L, Adamson GD, Keckstein J, Taylor HS, Abrao MS, et al World Endometriosis Society consensus on the classification of endometriosis. Hum Reprod. 2017 1 23;32(2):315–24. 10.1093/humrep/dew293 27920089

[38] Raffaelli R, Garzon S, Baggio S, Genna M, Pomini P, Lagana AS, et al Mesenteric vascular and nerve sparing surgery in laparoscopic segmental intestinal resection for deep infiltrating endometriosis. Eur J Obstet Gynecol Reprod Biol. 2018;221:214–219. 10.1016/j.ejogrb.2018.10.057 30415128

[39] Laganà AS, Vitale SG, Trovato MA, Palmara VI, Rapisarda AM, Granese R, et al Full-thickness excision versus shaving by laparoscopy for intestinal deep infiltrating endometriosis: rationale and potential treatment options.Biomed Res Int. 2016 10.1155/2016/3617179. PMC4989089 27579309

[40] Abo C, Moatassim S, Marty N, Saint Ghislain M, Huet E, Bridoux V, et al Postoperative complications after bowel endometriosis surgery by shaving, disc excision, or segmental resection: a three-arm comparative analysis of 364 consecutive cases. Fertil Steril. 2018;109(1):172–8. 10.1016/j.fertnstert.2017.10.001 29307394

[41] Nisolle M, Brichant G, Tebache L. Choosing the right technique for deep endometriosis. Best Pract Res Clin Obstet Gynaecol. 2019;pii: S1521-6934(18)30241-4. 10.1016/j.bpobgyn.2019.01.010 30824210

[42] HCUPnet. Healthcare Cost and Utilization Project. Rockville MD: US Agency for Healthcare Research and Quality [cited May 9, 2019]. Available from: https://hcupnet.ahrq.gov.

[43] Rehmer JM, Flyckt RL, Goodman LR, Falcone T. Management of Endometriomas. Obstet Gynecol Surv. 2019;74(4):232–240. 10.1097/OGX.0000000000000660 31344251

