# Supplementary material for: National trends in inpatient endometriosis admissions: Patients, procedures and outcomes, 2006−2015
Source: PLoS One. 2019 Sep 19;14(9):e0222889. doi: 10.1371/journal.pone.0222889 (PMC6752838; doi:10.1371/journal.pone.0222889)
Supplement: S1 Table — (DOCX) [file pone.0222889.s001.docx]

**S1 Table. Surgical procedures and corresponding ICD-9 diagnosis and procedure codes included in the analysis**

| Surgical Procedure | | ICD-9 Codes |
| --- | --- | --- |
| Hysterectomy | | |
| Subtotal abdominal hysterectomy | 68.3 | |
| Laparoscopic supracervical hysterectomy (LSH) | 68.31 | |
| Other and unspecified subtotal abdominal hysterectomy | 68.39 | |
| Total abdominal hysterectomy | 68.4 | |
| Laparoscopic total abdominal hysterectomy | 68.41 | |
| Other and unspecified total abdominal hysterectomy | 68.49 | |
| Vaginal hysterectomy | 68.5 | |
| Laparoscopically assisted vaginal hysterectomy (LAVH) | 68.51 | |
| Other and unspecified vaginal hysterectomy | 68.59 | |
| Radical abdominal hysterectomy | 68.6 | |
| Laparoscopic radical abdominal hysterectomy | 68.61 | |
| Other and unspecified radical abdominal hysterectomy | 68.69 | |
| Radical vaginal hysterectomy | 68.7 | |
| Laparoscopic radical vaginal hysterectomy (LVRH) | 68.71 | |
| Other and unspecified radical vaginal hysterectomy | 68.79 | |
| Pelvic evisceration | 68.8 | |
| Other and unspecified hysterectomy | 68.9 | |
|  |  | |
| Intervention on bladder | | |
| Open excision or destruction of other lesion or tissue of bladder | 57.59 | |
|  |  | |
| Laparoscopy | | |
| Laparoscopy; peritoneoscopy | 54.21 | |
| Other diagnostic procedures on abdominal region | 54.29 | |
|  |  | |
| Laparotomy | | |
| Exploratory laparotomy | 54.11 | |
| Reopening of recent laparotomy site | 54.12 | |
| Other laparotomy | 54.19 | |
| Other operations of abdominal region | 54.99 | |
| Other local excision or destruction of ovary | 65.29 | |
| Laparoscopic excision or destruction of lesion of uterus | 68.21 | |
| Other excision or destruction of lesion of uterus | 68.29 | |
| Excision or destruction of lesion of cul-de-sac | 70.32 | |
|  |  | |
| Oophorectomy | | |
| Oophorotomy | 65 | |
| Unilateral oophorectomy | 65.3 | |
| Laparoscopic unilateral oophorectomy | 65.31 | |
| Other unilateral oophorectomy | 65.39 | |
| Unilateral salpingo-oopherectomy | 65.4 | |
| Laparoscopic unilateral salpingo-oophorectomy | 65.41 | |
| Other unilateral salpingo-oophorectomy | 65.49 | |
| Bilateral oophorectomy | 65.5 | |
| Other removal of both ovaries at same operative episode | 65.51 | |
| Other removal of remaining ovary | 65.52 | |
| Laparoscopic removal of both ovaries at same operative episode | 65.53 | |
| Laparoscopic removal of remaining ovary | 65.54 | |
| Bilateral salpingo-oopherectomy | 65.6 | |
| Other removal of both ovaries and tubes at same operative episode | 65.61 | |
| Other removal of remaining ovary and tube | 65.62 | |
| Laparoscopic removal of both ovaries and tubes at same operative episode | 65.63 | |
| Laparoscopic removal of remaining ovary and tube | 65.64 | |
| Laparoscopic lysis of adhesions of ovary and fallopian tube | 65.81 | |
| Other lysis of adhesions of ovary and fallopian tube | 65.89 | |
|  |  | |
| Other excision/ablation | | |
| Endoscopic excision or destruction of lesion of duodenum | 45.3 | |
| Other local excision of lesion of duodenum | 45.31 | |
| Other destruction of lesion of duodenum | 45.32 | |
| Local excision of lesion or tissue of small intestine, except duodenum | 45.33 | |
| Other destruction of lesion of small intestine, except duodenum | 45.34 | |
| Excision of lesion or issue of large intestine | 45.41 | |
| Endoscopic destruction of other lesion or tissue of large intestine (includes both excision and destruction by endoscopic approach) | 45.43 | |
| Other destruction of lesion of large intestine (open approach) | 45.49 | |
| Excision or destruction of lesion or tissue of abdominal wall or umbilicus | 54.3 | |
| Excision or destruction of peritoneal tissue | 54.4 | |
| Other laparoscopic local excision or destruction of ovary | 65.25 | |
| Endometrial ablation | 68.23 | |
| Other excision or destruction of lesion of fallopian tube | 66.19 | |
| Excision or destruction of lesion of fallopian tube | 66.61 | |
|  |  | |
| Salpingectomy | | |
| Salpingotomy and salpingostomy | 66 | |
| Total unilateral salpingectomy | 66.4 | |
| Total bilateral salpingectomy | 66.5 | |

Abbreviation: ICD-9: International Classification of Diseases, 9^th^ edition
