# Supplementary material for: National trends in inpatient endometriosis admissions: Patients, procedures and outcomes, 2006−2015
Source: PLoS One. 2019 Sep 19;14(9):e0222889. doi: 10.1371/journal.pone.0222889 (PMC6752838; doi:10.1371/journal.pone.0222889)
Supplement: S2 Table — (DOCX) [file pone.0222889.s002.docx]

**S2 Table. Surgical complications and corresponding ICD-9 diagnosis and procedure codes included in the analysis**

| Complications | | ICD-9 Codes^*^ |
| --- | --- | --- |
| Medical/surgical complications | 996.x, 997.x, 998.x, 999.x | |
| Venous thromboembolism |  | |
| Pulmonary embolus | 415.11 | |
| Thrombophlebitis | 671.2, 453, 997.2 | |
| Menopausal symptoms | 627.2 | |
| Gastritis and duodenitis | 535 | |
| Nephropathy | 583.9 | |
| Infection |  | |
| Wound infection^†^ | 614.3, 614.4, 616.1, 998.5 | |
| Urinary tract infection | 599.0 | |
| Abscess | 614.9, 682, 639 | |
| Pyelonephritis | 599.80, 599.81 | |
| Cellulitis | 614.4 | |
| Sepsis | 995.91, 995.92, 996.64, 038, 790.7 | |
| Fever/pyrexia | 780.6 | |
| Vascular repair | 39 | |
| Damage to blood vessels | 904.9 | |
| Genitourinary tract injury |  | |
| Cystotomy | V44.50, 596.83 | |
| Ureteral injury (transection) | 867.2 | |
| Vesicovaginal fistula | 619.0 | |
| Ureterovaginal fistula | 593.82 | |
| Urinary retention | 788.2 | |
| Bladder atony | 596.4 | |
| Gastrointestinal tract injury |  | |
| Injury to gastrointestinal tract | 863.xx | |
| Anastomotic dehiscence/anastomotic leak syndrome | 997.49 | |
| Rectovaginal fistula | 619.1 | |
| Rectal stenosis | 569.2 | |
| Rectal perforation | 569.83 | |
| Bleeding |  | |
| Blood transfusion | 99.02, 99.04, V58.2 | |
| Hematoma | 665.7, 998.1, 998.12 | |
| Vascular injury | 997.7x | |
| Vaginal cuff dehiscence | 998.32 | |
| Nerve injury | 356 | |
| Repair to bladder | 57.8 | |
| Repair to ureter | 56.8 | |
| Repair to bowel | 46.7 | |
| Other complications^‡^ | E870, E878 | |

Abbreviation: ICD-9: International Classification of Diseases, 9^th^ edition

* Included both diagnosis and procedure ICD-9 codes.

^†^ Wound infections included acute parametritis and pelvic cellulitis, chronic or unspecified parametritis and pelvic cellulitis, vaginitis and vulvovaginitis, postoperative infection.

^‡^ Other complications included accidental cut, puncture, perforation or hemorrhage during medical care and surgical procedures as the cause of abnormal reaction of patient or later complication.
